# Neonatal atypical hemolytic uremic syndrome from a factor H mutation treated with eculizumab 

**DOI:** 10.5414/CN108532

**Published:** 2015-03-27

**Authors:** Sheena Sharma, Madhura Pradhan, Kevin E.C. Meyers, Krish Le Palma, Benjamin L. Laskin

**Affiliations:** 1 Division of Nephrology and; 2 Department of Pharmacy Services, The Children’s Hospital of Philadelphia, Philadelphia, PA, USA

**Keywords:** atypical hemolytic uremic syndrome (aHUS), neonates

## Abstract

Background: Atypical hemolytic uremic syndrome (aHUS) results from an inherited dysregulation of the alternative complement pathway leading to thrombotic microangiopathy consisting of hemolytic anemia, thrombocytopenia, and renal injury. The complement inhibitor eculizumab is an approved treatment, but its reported use in neonates – who have an inherently high risk of infection – is limited. Case diagnosis/treatment: A 28-day-old female presented with gross hematuria and hypertension. aHUS was suspected based on anemia with schistocytes, thrombocytopenia, low C3, and acute kidney injury requiring peritoneal dialysis. A septic work-up initiated on day 2 for hypothermia and respiratory failure was negative. There was no improvement after 6 days of plasma therapy. Despite being < 6 weeks old she was vaccinated with pneumococcal-13 conjugate, meningococcal (groups C and Y) polysaccharide, and *Haemophilus b* tetanus toxoid conjugate vaccines and started on penicillin prophylaxis. After 1 dose of eculizumab 300 mg, dialysis was discontinued and her hematological parameters improved. Genetic testing revealed a complement factor H mutation. After 11 months of follow-up, she remains on eculizumab and penicillin without recurrence of aHUS or any infectious complications. Conclusions: Eculizumab is a safe and effective treatment option for aHUS even in neonates at high risk for infection.

## Introduction 

Atypical hemolytic uremic syndrome (aHUS) occurs when uncontrolled activation of the alternative complement pathway causes systemic endothelial damage via the membrane attack complex [1]. Approximately 5 – 10% of patients with hemolytic anemia, thrombocytopenia, and acute kidney injury have aHUS, of which, 50 – 60% are associated with genetic mutations encoding complement factors [2]. 

Eculizumab is approved to treat aHUS [3]. Because eculizumab inhibits formation of the membrane attack complex, patients are at risk for infections from encapsulated bacteria [4]. Patients receiving eculizumab require vaccination and/or antibiotic prophylaxis against *Neisseria meningitidis*, *Streptococcus pneumoniae*, and *Haemophilus influenza* [4]. Although eculizumab is approved for children, little is known about the dosing schedule in those < 5 kg and the infectious risk of treating very young patients, who have an inherently high risk of infection. 

Only two reports have described the use of eculizumab in neonates with aHUS [1, 5]. We report a 28-day-old neonate with aHUS who was treated with eculizumab, stressing the immunization and infection management decisions, and in addition, review the published literature regarding the treatment of this condition in neonates and infants. 

## Case report 

A 28-day-old female, born at 41 weeks gestation (birth weight 3.3 kg) to non-consanguineous parents, presented with three episodes of gross hematuria. Antenatal ultrasound was normal. There was no family history of kidney disease and she was taking no medications. 

Except for a blood pressure of 127/65 mmHg, physical examination was normal. Laboratory studies showed: hemoglobin 7.3 g/dL, platelet count 54,000/µL, blood urea nitrogen 60 mg/dL, and serum creatinine concentration of 2.0 mg/dL. Urinalysis showed large blood and 4+ protein. Lactate dehydrogenase was 4,271 units/L (normal 600 – 2,100 units/L) with 1+ schistocytes on peripheral smear. Doppler ultrasound revealed diffusely echogenic kidneys and no thrombosis. Serum C3 concentration was 25 mg/dL (normal 67 – 161 mg/dL) and CH50 was 154 units/mL (normal 104 – 356 units/mL). Normal serum homocysteine and methylmalonic acid concentrations made cobalamin C deficiency unlikely. 

Daily plasma infusions (15 – 20 mL/kg/day) were started on hospital day 2 and peritoneal dialysis on day 3 due to worsening oliguria. Shortly thereafter, she experienced acute respiratory failure and hypothermia, resulting in a septic work-up and cessation of dialysis. The C-reactive protein was 1.5 mg/dL (normal 0 – 0.9 mg/dL) and there were 5.3% bands with a normal peripheral white cell count. Blood, cerebrospinal, and peritoneal fluid cultures were obtained and vancomycin, cefepime, and metronidazole were administered until cultures were negative. An echocardiogram showed moderately diminished biventricular function. A head ultrasound was normal. 

After intubation, milrinone and dobutamine were started and peritoneal dialysis was resumed. Despite daily plasma infusions, she required an additional eight red blood cell and three platelet transfusions. Plasmapheresis was added on day 6 with little clinical improvement. Therefore, eculizumab was infused on day 7. The pneumococcal-13 conjugate, meningococcal (groups C and Y) polysaccharide, and *Haemophilus b* tetanus toxoid conjugate vaccines were administered within 24 hours of receiving eculizumab. Despite weighing < 5 kg, eculizumab was dosed for patients weighing 5 – 10 kg (300 mg intravenous weekly for two doses, followed by 300 mg every 3 weeks), according to internal data from Alexion Pharmaceuticals and the two prior reports [1, 5]. The patient was started on prophylactic ampicillin and transitioned to penicillin VK. 

Within 4 days peritoneal dialysis was stopped, and within 5 days hematological markers improved. The CH50 was 0 units/mL 2 days after eculizumab dosing. ADAMST13 had 76% activity (normal ≥ 67%), factor I level was 37.1 µg/mL (normal 29.3 – 58.5 µg/mL), factor H level was 85 µg/mL (normal 160 – 412 µg/mL) and factor H auto-antibodies were not detected. Genetic testing revealed a missense mutation in factor H (exon 22) and a variant of unknown significance (exon 8). At 12 months of age, she is maintained on eculizumab 300 mg every 3 weeks, penicillin VK 125 mg twice daily, and propranolol for a history of supraventricular tachycardia. She recently had a mediport placed for ease of drug administration. Her serum creatinine concentration is 0.2 mg/dL and she has no proteinuria and a normal blood pressure. 

## Discussion 

We present a 28-day-old neonate with aHUS who received eculizumab after failing plasma therapy. Her acute decompensation was cardiogenic based on the echocardiogram findings. Nevertheless, there was considerable concern regarding administering eculizumab to an unvaccinated infant with possible sepsis. 

Plasma therapy is considered the treatment of choice for aHUS, but there are reports of worse outcomes when used as initial treatment and the risks of catheter placement for plasma exchange must be considered [6]. Therefore, with the approval of eculizumab, this drug may become first-line therapy [1]. 

Two prior cases of neonates (< 5 kg) receiving eculizumab have been published (Table 1). An 11-day-old received eculizumab after presenting with cardiogenic shock and a negative septic evaluation. Vaccination against *Neisseria meningitidis* occurred at 6 months of age (serotypes not reported), but the patient did receive prophylactic oral penicillin. Notably, she had *Klebsiella pneumoniae* sepsis at 6 months [1]. A 28-day-old with no concerns for infection received eculizumab along with the meningococcal vaccine (serotypes not reported) and amoxicillin prophylaxis. The patient had 3 infectious episodes while receiving eculizumab: respiratory syncytial virus, diarrhea, and rotavirus [5]. 

The optimal dosing regimen for eculizumab in young infants remains unknown (Table 1) [1, 5]. However, our case, along with the other two reported neonates, supports maintenance dosing of 300 mg every 3 weeks. In the patient treated by Ariceta et al. [5], eculizumab dosing of 150 mg resulted in a sub-therapeutic eculizumab level of 19.1 µg/mL (goal > 50 – 100 µg/mL) and hematological relapse that responded to dosing at 300 mg. 

Because eculizumab levels are only available in one laboratory in the United States (Cambridge Biomedical, Inc., Boston, MA, USA) and turn-around time can be > 1 week, there is a need for markers that can optimize dosing [6]. In 6 children diagnosed with post-hematopoietic stem cell transplant thrombotic microangiopathy, a very low CH50 level correlated with therapeutic eculizumab drug levels and a favorable clinical response [3]. In our patient, the CH50 level was zero 2 days after receiving eculizumab, correlating with remission of disease. However, others have shown that despite a low CH50 and “therapeutic” eculizumab level, plasma terminal complement levels (C5b-9) can remain high, which suggests ongoing complement activation [7]. Therefore, more research is needed to determine which markers reliably indicate therapeutic complement blockade [6]. 

It is also unknown if eculizumab can be discontinued in patients with aHUS. In a recent study by Ardissino et al. [8], 10 patients with aHUS successfully treated with eculizumab stopped therapy while microscopic hematuria was monitored at home as a marker of recurrence. Of their 10 patients, 3 had recurrence of disease within 6 weeks of stopping eculizumab; however all achieved complete remission with re-initiation of eculizumab. Due to the limited evidence regarding stopping therapy in young children, we are currently favoring life-long therapy for our patient. 

The risks of prolonged eculizumab therapy include immunosuppression, the need for prophylactic antibiotics, repeated vascular access in a population potentially at high risk for chronic kidney disease, and the very high financial costs of therapy. The average wholesale price (AWP) of a 300 mg vial of eculizumab is currently listed at $7,196.40 [9]. Based on an every 3 week dosing schedule, the annual cost to our patient, excluding drug administration, would therefore be at least $125,000. Some have supported liver transplantation as a curative option for aHUS, but the risks and benefits of this procedure need to be considered on a case by case basis [10]. 

In neonates with aHUS, we support using eculizumab with simultaneous initiation of antibiotic prophylaxis. The manufacturer recommends vaccination occur within 2 weeks of starting eculizumab. We recommend vaccines against pneumococcus, meningococcus (serotypes ACYW135) and *Haemophilus influenzae* type b that are approved in the United States for infants ≥ 6 weeks of age (Table 2) [14]. Meningococcal serogroups vary based on age and geographic distribution. Over half of infant meningococcal cases in the United States are caused by serogroup B, however, the recently developed serotype B vaccine will likely only be available for patients 10 – 25 years of age [11]. The manufacturer also recommends antibiotic prophylaxis for 2 weeks following meningococcal vaccination or indefinitely in patients who cannot be vaccinated; however, the type of antibiotic is not mentioned [12]. We suggest using penicillin based on sickle cell disease patients who require antibiotic prophylaxis for functional asplenia [13]. Because available neonatal meningococcal vaccines do not cover all disease-causing serotypes, we support continuing antibiotics indefinitely [14]. While our case supports a maintenance dosing regimen of 300 mg every 3 weeks, more research is needed to determine the most cost-effective dosing, frequency, and duration of therapy in the smallest patients, potentially guided by markers of complement activity [1, 2, 5]. 

## Conflict of interest 

SS participated in a dinner case presentation sponsored by Alexion. BLL is co-inventor on the following patent application, filed September 16, 2014: “Compositions and Methods for Treatment of HSCT-Associated Thrombotic Microangiopathy” application number PCT/US2014/055922. No other authors had a conflict of interest to disclose. 

**Table 1:** Reported cases of eculizumab treatment in children < 10 kg with atypical hemolytic uremic syndrome.

Reference	Patient age at presentation	Eculizumab dosing	Antibiotic prophylaxis	Immunizations	Most recent follow-up
Patient weight < 5 kg					
Michaux et al . [1]	11 days (unclear which day drug started)	300 mg weekly for 2 doses then, every 2 weeks for 2 months then, every 3 weeks	Penicillin VK until vaccination	Meningococcal vaccine* given at 6 months of age	At 27 months old, normal renal function, no proteinuria, normal blood pressure
Ariceta et al. [5]	28 days (39 days old when started eculizumab)	300 mg for 1 dose then, 150 mg for 1 dose (relapse) then, 300 mg every 3 weeks	Amoxicillin (duration NR)	Meningococcal vaccine* before eculizumab	At 14 months old, normal renal function, proteinuria (urine protein/creatinine ratio 1 mg/g), normal blood pressure
Current case	28 days (35 days old when started eculizumab)	300 mg weekly for 2 doses then, every 3 weeks	Ampicillin then penicillin VK ongoing	Pneumococcal-13 conjugate, meningococcal CY polysaccharide, and *Haemophilus b* tetanus toxoid within 24 hours of eculizumab	At 12 months old, normal renal function, no proteinuria, normal blood pressure
Patient weight 5 – 10 kg					
Ohta et al. [14]	4 months	300 mg weekly for 2 doses then, every 3 weeks	Cefdinir ongoing	Meningococcal ACYW-135 polysaccharide after eculizumab	At 22 month old, serum creatinine 0.33 mg/dL, no proteinuria
Gilbert et al . [7]	4 months	300 mg weekly for 2 doses then, every 3 weeks	NR	NR	NR
Besbas et al. [2]	6 months	300 mg weekly for 3 weeks then, 600 mg every 2 weeks	NR	NR	At 20 months old, normal renal function
Kim et al. [15]	7 months	300 mg weekly for 2 doses then, every 3 weeks	NR	Meningococcal ACYW-135 2 weeks before eculizumab	At 18 months old, creatinine eGFR 42 mL/min/1.73m^2^
Giordano et al. [16]	1 year	300 mg weekly for 4 weeks then, every 2 weeks for 2 months then, every 3 weeks	NR	Meningococcal vaccine* before eculizumab	At 23 months old, creatinine 0.51 mg/dL, proteinuria (urine protein/creatinine ratio 0.47)

NR = not reported; eGFR = estimated glomerular filtration rate. *Meningococcal serotypes not reported.

**Table 2:** Vaccine and antibiotic recommendations for children receiving eculizumab [12, 13, 17, 18].

Meningococcal vaccinations available for children (United States)	Dosing schedule
Hib-MenCY	≥ 6 weeks of age, 4 dose series at 2, 4, 6, and 12 – 15 months of age
MenACWY-CRM	≥ 2 – 7 months of age, 4 dose series at 2, 4, 6, and 12 months of age
	≥ 7 – 23 months of age, 2 dose series 3 months apart with 2^nd^ dose given at > 12 months of age
MenACWY-D	≥ 9 months of age, 2 dose series 3 months apart

The eculizumab package insert recommends antibiotic prophylaxis for 2 weeks only if eculizumab is started < 2 weeks after the meningococcal vaccination. In children, we recommend indefinite antibiotic prophylaxis during eculizumab treatment and meningococcal, pneumococcal, and *Haemophilus b* immunizations in patients ≥ 6 weeks. Antibiotic prophylaxis options: Ampicillin: 50 mg/kg/dose IV every 24 hours, maximum 1,000 mg/dose. Penicillin VK: < 5 years of age, 125 mg PO twice daily; ≥ 5 years of age, 250 mg twice daily. Amoxicillin: 20 mg/kg/day PO divided once or twice daily, maximum 500 mg/day. Erythromycin: (if penicillin-allergic), < 3 years of age, 125 mg PO twice daily; ≥ 3 years of age, 250 mg PO twice daily. Hib-MenCY = meningococcal polysaccharide (groups C/Y) and *Haemophilus b* tetanus toxoid conjugate vaccine; MenACWY-CRM = meningococcal (groups A/C/Y and W-135) oligosaccharide conjugated to CRM197 protein vaccine; MenACWY-D = meningococcal (groups A/C/Y and W-135) polysaccharide diphtheria conjugate vaccine; IV = intravenous; PO = oral.
